# Intra- and Inter-Operator Reliability of Internal Jugular Vein Ultrasound Image Acquisition by Ultrasound-Naive Nurses

**DOI:** 10.3390/healthcare14193272

**Published:** 2026-10-02

**Authors:** Yukie Tamura, Kazumi Ishii, Miharu Matsumoto, Hiromitsu Kobayashi

**Affiliations:** 1 Faculty of Nursing, Ishikawa Prefectural Nursing University, 1-1, Gakuendai, Kahoku 929-1210, Ishikawa, Japan; tamura@ishikawa-nu.ac.jp (Y.T.);; 2 Department of Health Sciences, Kyushu University, 3-1-1, Maidashi, Higashi-Ku, Fukuoka 812-8582, Fukuoka, Japan

**Keywords:** heart failure, internal jugular vein, ultrasonography, image acquisition, reliability, nurses, point-of-care ultrasound

## Abstract

**Highlights:**

**What are the main findings?**

- Following preparatory training, two nurses with no previous clinical ultrasound experience successfully acquired short-axis IJV images from all healthy participants during quiet breathing and the Valsalva maneuver.
- Intra-operator reliability ranged from good to excellent, whereas inter-operator reliability based on a single acquisition ranged from moderate to excellent.

**What are the implications of the main findings?**

- These findings support the technical feasibility of IJV ultrasound image acquisition by nurses without previous clinical ultrasound experience after preparatory training.

**Abstract:**

**Background:** Venous congestion assessment is essential in heart failure management. Inferior vena cava (IVC) ultrasonography has been widely used to determine fluid status but may not always provide satisfactory images. Hence, internal jugular vein (IJV) ultrasonography has been proposed as an alternative; however, the reliability of IJV ultrasound image acquisition by nurses without previous clinical ultrasound experience remains unclear. This study evaluated the intra- and inter-operator reliability of IJV ultrasound image acquisition by nurses without previous clinical ultrasound experience. **Methods:** Two nurses without prior clinical ultrasound experience acquired right IJV ultrasound images after preparatory training. Short-axis cine loops were obtained during quiet breathing and the Valsalva maneuver in healthy adults. A single investigator measured the major and minor diameters from selected still frames. Intra- and inter-operator reliability were then assessed using intraclass correlation coefficients (ICCs), after which agreement was evaluated by Bland–Altman analysis. **Results:** Both nurses successfully acquired IJV images suitable for subsequent quantitative analysis from all participants. Intra-operator reliability ranged from good to excellent, with ICC (1, 1) values of 0.765–0.967. Meanwhile, inter-operator reliability ranged from moderate to excellent, with ICC (2, 1) values of 0.629–0.916. Bland–Altman analysis revealed fixed bias in three parameters but no proportional bias. Absolute bias was <1 mm for all measurements. **Conclusions:** Following preparatory training, nurses without prior clinical ultrasound experience were able to acquire IJV ultrasound images with moderate-to-excellent inter-operator and good-to-excellent intra-operator relative reliability.

## 1. Introduction

Heart failure remains a major global public health concern, with its prevalence continuing to increase with population aging [1]. Chronic heart failure is a progressive condition that substantially impacts quality of life and prognosis. Patients with chronic heart failure have high rates of hospital readmissions, which impose a considerable economic burden on healthcare systems [1,2]. Furthermore, during worsening heart failure, hemodynamic congestion may precede overt clinical signs and symptoms, such as dyspnea and peripheral edema [3]. Therefore, early detection of congestion and timely intervention are crucial for reducing the risk of heart failure-related hospital readmissions [4].

Nurses play an important role in detecting early signs of worsening heart failure through ongoing patient monitoring and assessment across various healthcare settings, including inpatient wards, outpatient clinics, and home care settings. Many patients with heart failure are older adults with multiple comorbidities [5]. Frailty and cognitive impairment are also common among older patients with heart failure, which may contribute to atypical clinical presentations or difficulty in accurately reporting changes in their condition [6,7]. Consequently, early detection of worsening heart failure can be challenging [8], highlighting the need for simple, noninvasive indicators that enable nurses to objectively assess changes in patients’ clinical status.

One noninvasive method for assessing congestion in patients with heart failure is the ultrasonographic measurement of inferior vena cava (IVC) diameter and its respiratory variation. IVC diameter and its respiratory variation have been used to estimate central venous pressure and are widely used as clinical indicators of congestion in patients with heart failure [9,10,11]. However, the IVC can be difficult to visualize owing to various factors, such as body habitus, bowel gas, and intrathoracic air [12]. Previous studies have reported that IVC measurements could not be obtained in approximately 10% of cases [10,13].

Ultrasonographic assessment of the internal jugular vein (IJV) has attracted attention as a potential alternative or complementary approach when IVC assessment is difficult [14,15,16]. Given its superficial location in the neck, the IJV is less affected by abdominal factors that can limit IVC visualization.

Previous studies have reported that IJV diameter, cross-sectional area, respiratory variations, and variations during the Valsalva maneuver were associated with central venous pressure and right atrial pressure [17,18]. In outpatients with heart failure, a lower ratio of maximum IJV diameter during the Valsalva maneuver to that at rest was associated with an increased risk of death or hospitalization for heart failure [19]. In addition, in patients with acute decompensated heart failure, a smaller percentage increase in IJV cross-sectional area during the Valsalva maneuver at discharge was associated with an increased risk of 30-day readmission [20]. These findings suggest that IJV ultrasound parameters may be useful adjunctive indicators in the noninvasive assessment of congestion in patients with heart failure. However, considering that the IJV is superficial and easily compressed, transducer pressure may affect the acquired images and resulting measurements [21,22,23]. Lucas et al. reported that, in ultrasonographic measurements of central vein diameter, random measurement errors showed greater variability during image acquisition than during interpretation of the acquired images [24]. Furthermore, IJV diameter may be influenced by body position, respiratory conditions, and measurement site [25,26].

Although various IJV ultrasound parameters, such as diameter, cross-sectional area, variations during the Valsalva maneuver, and IJV vertical height, have been investigated, the measurement procedures and parameters used have yet to be sufficiently standardized [15,27]. Furthermore, only a handful of studies have examined the reliability of ultrasonographic IJV measurements. Although some previous studies have reported good inter-operator reliability using intraclass correlation coefficients (ICCs) [17,18], the professional backgrounds and ultrasound experience of the operators have not been clearly described. Nurses must continuously monitor changes in congestion in patients with heart failure. To this end, determining whether nurses can reliably acquire IJV ultrasound images is an essential step toward the clinical application of this method.

Therefore, as a preliminary study for future clinical application in patients with heart failure, the present study evaluated the intra- and inter-operator reliability of IJV ultrasound image acquisition by nurses with no previous clinical ultrasound experience using ICCs and Bland–Altman analysis.

## 2. Methods

### 2.1. Participants

This study enrolled a total of 17 healthy adults aged 18 years or older (mean age, 19.9 ± 1.3 years; mean body mass, 51.1 ± 5.7 kg; mean height, 160.0 ± 5.7 cm; and mean body mass index, 20.0 ± 2.0 kg/m^2^; Table 1). Individuals with medical conditions that could affect intravascular volume, as well as pregnant women, were excluded. Participants were instructed to refrain from eating or drinking for 1 h before the ultrasound examination. The study was conducted in accordance with the Declaration of Helsinki and approved by the Ethics Committee of Ishikawa Prefectural Nursing University (approval number: 2023-437-2; date of approval: 12 January 2024). Before participation, the researchers explained the purpose of the study and the procedures involved to all participants and obtained oral and written informed consent.

### 2.2. Ultrasound Equipment and Operators

Ultrasound examinations were performed using a portable ultrasound device (Vscan Air; GE HealthCare Japan Co., Ltd., Tokyo, Japan). A linear-array transducer with a frequency range of 7.5–12 MHz was used for IJV image acquisition.

IJV ultrasound images were acquired by two nurses (Operators A and B) with no previous clinical experience in ultrasonography. Before the study, both operators were trained for approximately 90 min in device manipulation, IJV identification, transducer pressure adjustment, and ultrasound image acquisition with reference to procedures described in previous studies [21,22,23,28]. Adequate visualization of the IJV was defined as meeting the following criteria: absence of compression by the transducer, inclusion of the entire IJV contour within the ultrasound image, and visualization of the adjacent carotid artery and sternocleidomastoid muscle. These criteria were explained to each operator during the training. Each operator subsequently practiced the study procedures on approximately 10 healthy adults over a one-week period. Data collection was initiated after a researcher experienced in IJV ultrasonography reviewed the acquired images and confirmed that they met these criteria.

### 2.3. Procedure

This study was conducted between August and December 2024. The temperature in the experimental room was kept at 23–24 °C. Participants were placed in the supine position without a pillow, and their heads were kept in a neutral position. They rested for 1 min to allow their breathing to stabilize.

A linear-array transducer was gently placed over the right sternocleidomastoid muscle at the level of the thyroid cartilage to obtain a transverse short-axis view of the right IJV. A generous amount of ultrasound gel was applied to minimize vein compression. The imaging depth was set at 40 mm, and the gain was adjusted by each operator to clearly delineate the vessel wall–lumen interface.

During quiet breathing, participants were instructed to breathe naturally, and a 10-s cine loop that included at least one complete respiratory cycle from inspiration to expiration was recorded. During the Valsalva maneuver, participants were instructed to hold their breath and bear down by contracting the lower abdominal muscles. A 10-s cine loop was recorded to capture the period during which IJV distension was observed and its diameter stabilized.

Each operator acquired three cine loops from each participant. After each acquisition, the transducer was removed from the skin. To avoid consecutive acquisitions by the same operator, Operators A and B alternated image acquisition. Each operator acquired the images independently without observing the other operator’s technique.

### 2.4. Image Analysis

Given the present study’s focus on the reproducibility of ultrasound image acquisition by the operators, frame selection and diameter measurements were performed by a single researcher. The reliability of the image analysis itself was not evaluated.

From each cine loop acquired during quiet breathing, still frames of the IJV reaching its minimum and maximum sizes were selected. From each cine loop acquired during the Valsalva maneuver, a still frame of when IJV expansion had stabilized was selected. These three time points were referred to as the minimum during quiet breathing, and the maximum during quiet breathing and during the Valsalva maneuver, respectively.

**Figure 1:**
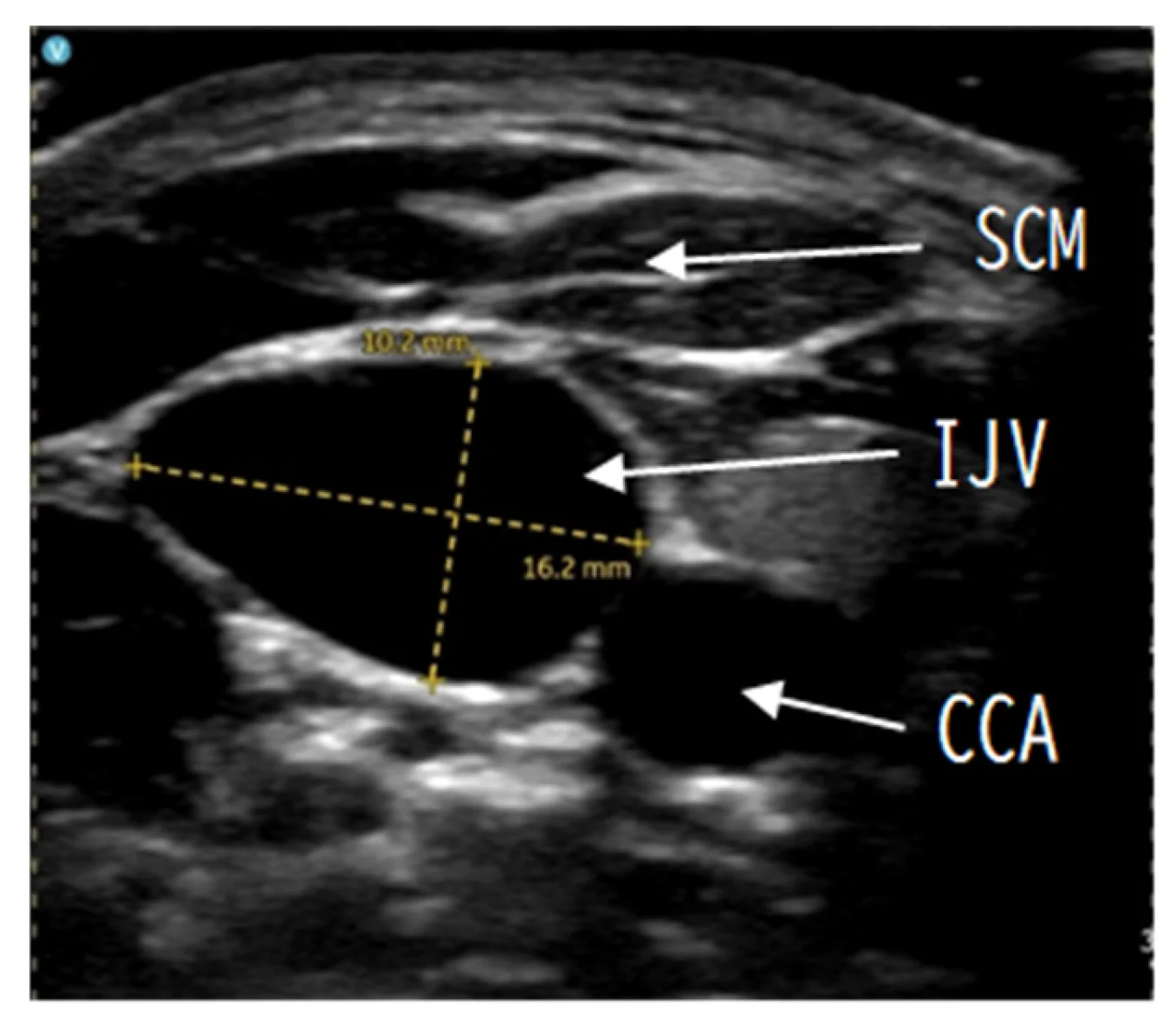
Example image of the internal jugular vein. SCM: sternocleidomastoid muscle; IJV: internal jugular vein; CCA: common carotid artery.

In each selected frame, the longest diameter of the IJV cross-section was considered the major diameter, whereas the diameter perpendicular to the major diameter was considered the minor diameter (Figure 1). Both diameters were measured using the two-point caliper function of the Vscan Air device.

Frame selection and diameter measurements were performed with the images presented in random order, with the researcher being blinded to the operator, participant, and trial number. After completing all measurements, an identification key was used to match each measurement with the corresponding operator, participant, and trial number.

### 2.5. Statistical Analysis

Sample size was calculated using the method described by Walter et al. [29]. An expected ICC of 0.80 and a minimum acceptable ICC of 0.50 were specified a priori based on commonly used interpretive criteria for ICC values [30]. Assuming three repeated measurements per participant, a significance level of 0.05, and a statistical power of 0.80, the required sample size was 15 participants.

The distributions of continuous variables were examined for normality using the Shapiro–Wilk test, together with an assessment of skewness and outliers. Although one variable showed a significant result in the Shapiro–Wilk test, the overall assessment of the distributions did not indicate substantial non-normality; therefore, continuous variables are presented as mean ± standard deviation (SD).

For each measurement parameter, the mean and SD were calculated separately for each operator. Intra-operator reliability was assessed using ICC (1, 1) based on the three repeated measurements for each participant. Inter-operator reliability was assessed using ICC (2, 1) separately for each of the three measurement trials. The 95% confidence interval (CI) was calculated for each ICC estimate.

Agreement between Operators A and B was further assessed using Bland–Altman analysis. The mean difference (bias) and the 95% limits of agreement (LoA) were calculated. Fixed bias was considered present when the 95% CI of the mean difference did not include zero. Proportional bias was assessed using linear regression of the differences between measurements derived from images acquired by the two operators against their corresponding means.

All statistical analyses were performed using IBM SPSS Statistics version 29.0 (IBM Corp., Armonk, NY, USA). A *p* value < 0.05 was considered statistically significant.

## 3. Results

### 3.1. Image Acquisition Feasibility and Descriptive Statistics

Both operators successfully acquired short-axis images of the right IJV in all participants during quiet breathing and the Valsalva maneuver.

The mean and SD of each diameter measurement are summarized in Table 2. At the minimum during quiet breathing, the major diameter was 13.4 ± 2.6 mm and 12.8 ± 2.7 mm, whereas the minor diameter was 7.5 ± 2.2 mm and 7.6 ± 2.4 mm for Operators A and B, respectively. At the maximum during quiet breathing, the major diameter was 14.8 ± 2.7 mm and 14.1 ± 2.7 mm, whereas the minor diameter was 8.7 ± 2.0 mm and 8.9 ± 2.2 mm for Operators A and B, respectively. During the Valsalva maneuver, the major diameter was 17.8 ± 4.1 mm and 16.9 ± 3.9 mm, whereas the minor diameter was 11.1 ± 2.4 mm and 11.9 ± 2.3 mm for Operators A and B, respectively.

### 3.2. Intra-Operator Reliability

Based on the results for the intra-operator reliability analysis (Table 3), ICC (1, 1) values ranged from 0.765 to 0.967 and from 0.881 to 0.941 for Operators A and B, respectively.

### 3.3. Inter-Operator Reliability

Based on the results of the inter-operator reliability analysis (Table 4) for each of the three measurement trials, ICC (2, 1) values ranged from 0.699 to 0.842 and from 0.642 to 0.849 for the major and minor diameters at the minimum during quiet breathing, respectively. At the maximum during quiet breathing, ICC (2, 1) values ranged from 0.799 to 0.916 and from 0.683 to 0.878 for the major and minor diameters, respectively. During the Valsalva maneuver, ICC (2, 1) values ranged from 0.838 to 0.903 and from 0.629 to 0.863 for the major and minor diameters, respectively.

### 3.4. Bland–Altman Analysis

**Figure 2:**
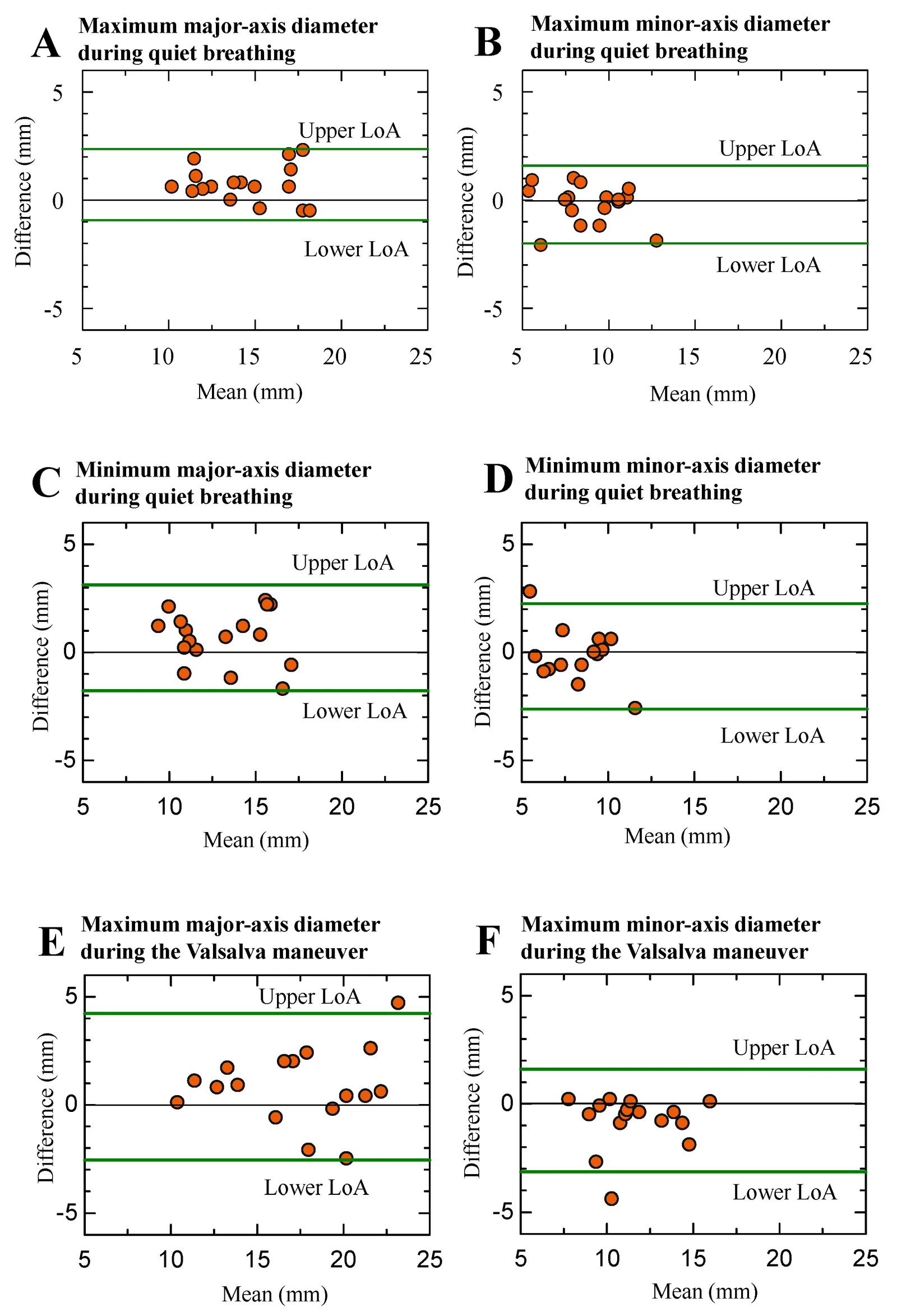
Bland–Altman plots showing the agreement between internal jugular vein (IJV) diameter measurements obtained from ultrasound images acquired by Operators A and B. (**A**) Maximum major diameter during quiet breathing. (**B**) Maximum minor diameter during quiet breathing. (**C**) Minimum major diameter during quiet breathing. (**D**) Minimum minor diameter during quiet breathing. (**E**) Maximum major diameter during the Valsalva maneuver. (**F**) Maximum minor diameter during the Valsalva maneuver.

The results of the Bland–Altman analysis are presented in Table 5 and Figure 2. At the minimum, during quiet breathing, the bias was 0.68 and −0.19 mm for the major and minor diameters, respectively. At the maximum during quiet breathing, the bias was 0.72 and −0.21 mm for the major and minor diameters, respectively. During the Valsalva maneuver, the bias was 0.84 and −0.77 mm for the major and minor diameters, respectively. Under all measurement conditions, the bias was positive for the major diameter but negative for the minor diameter.

At the minimum during quiet breathing, the 95% LoA ranged from −1.78 to 3.13 mm and from −2.63 to 2.25 mm for the major and minor diameters, respectively. At the maximum during quiet breathing, the 95% LoA ranged from −0.93 to 2.38 mm and from −2.01 to 1.59 mm for the major and minor diameters, respectively. During the Valsalva maneuver, the 95% LoA ranged from −2.55 to 4.23 mm and from −3.14 to 1.60 mm for the major and minor diameters, respectively.

A fixed bias for the major diameter was observed at the minimum and maximum during quiet breathing, whereas that for the minor diameter was observed during the Valsalva maneuver. No proportional bias was observed in any measurement condition. The absolute value of the bias was <1 mm under all measurement conditions.

The central line represents zero difference, whereas the upper and lower lines represent the 95% limits of agreement (LoA). Differences were calculated by subtracting the measurement derived from the image acquired by Operator A from that derived from the image acquired by Operator B.

## 4. Discussion

The present study found that both operators were able to acquire short-axis images of the IJV for all participants across all conditions. Furthermore, all images were suitable for subsequent quantitative analysis. Several studies have suggested that the IJV may serve as an alternative ultrasound imaging site when visualization of the IVC is difficult [14,15,31]. Indeed, a previous study by our group involving patients with chronic heart failure receiving home care reported that the IJV could be visualized for patients in whom IVC imaging was difficult [32]. These findings suggest that the IJV may be easier to visualize than the IVC, at least with respect to ultrasound image acquisition.

Our results showed that intra-operator ICC (1, 1) values ranged from 0.765 to 0.967, whereas inter-operator ICC (2, 1) values ranged from 0.629 to 0.916. Fields et al. [33], who evaluated the inter-operator reliability of maximum and minimum IVC diameter measurements obtained by clinicians in emergency patients, reported ICCs of 0.81 and 0.77, respectively. Moreover, Blanca et al. [34] reported an inter-operator ICC of 0.655 for IVC diameter measurements in healthy adults performed by physicians with experience in point-of-care ultrasound (POCUS). Although the operators in the present study were nurses without previous clinical ultrasound experience, the inter-operator reliability of IJV ultrasound image acquisition was generally equivalent to or higher than the ICC values reported in previous studies on IVC diameter measurements. As discussed earlier, previous studies on the reliability of IVC diameter measurements have primarily examined inter-operator reliability. Therefore, the results presented in this study regarding intra-operator reliability in IJV image acquisition are novel.

The ICCs observed in the present study indicate good-to-excellent relative reliability for most IJV diameter parameters. However, ICC values are influenced by between-subject variability and therefore do not directly indicate the absolute agreement between repeated measurements. Thus, high ICC values alone should not be interpreted as evidence of sufficiently close agreement for clinical use. In the present study, the ICC findings should be interpreted together with the Bland–Altman analysis, which provides complementary information regarding systematic bias and the variability of differences between individual acquisitions.

The Bland–Altman analysis revealed fixed bias in some parameters; however, the mean biases were small, indicating limited systematic differences between operators. Nevertheless, the relatively wide limits of agreement indicate that differences of several millimeters may occur in individual acquisitions. Because clinically acceptable limits of agreement were not defined a priori, the present study cannot determine whether the observed limits of agreement are acceptable for clinical decision-making.

In recent years, the use of IJV ultrasound to evaluate congestion in patients with heart failure has been increasing, particularly at the bedside and in outpatient settings [35,36]. The present study provides preliminary evidence regarding the reliability of IJV ultrasound image acquisition by nurses without previous clinical ultrasound experience.

The scope of practice for nurse-performed ultrasonography varies across countries and healthcare systems. Therefore, regulatory and professional requirements for the clinical implementation of nurse-performed IJV ultrasound should be considered separately from the technical feasibility of IJV image acquisition demonstrated in the present study. If incorporated into nursing practice within the appropriate regulatory and training framework, IJV ultrasound may provide nurses with an additional objective method for assessing venous congestion, particularly when IVC imaging is difficult.

This study has several limitations. First, it was conducted on healthy adults in a controlled laboratory setting, where body and head positions, as well as transducer placement, could be standardized. Therefore, it is unclear whether a similar level of reliability can be achieved in patients with heart failure or in real-world clinical environments, such as hospital wards and home care settings. Although predefined criteria were used to determine whether the acquired images were adequate, no quantitative proficiency threshold or standardized competency assessment was established for completion of the training. Therefore, the training protocol used in this study may not be directly generalizable to other nurses or clinical settings.

In addition, only two nurse operators were included in this preliminary study. Therefore, the findings may not represent the variability in image acquisition among nurses in general and should be interpreted as preliminary.

Furthermore, although this study evaluated the reliability of IJV ultrasound image acquisition by the operators, still-frame selection from the acquired cine loops and diameter measurements were performed by a single researcher. Therefore, the reliability of still-frame selection and diameter measurement performed by nurses themselves was not evaluated. Lucas et al. [24] reported that in POCUS-based measurement of central venous diameter, variability arising from image acquisition had a greater impact on measurement error than did image quantification. A similar effect may also occur in IJV imaging. Therefore, the reliability results from this study, which were limited to image acquisition, are of some significance.

## 5. Conclusions

The present study provides preliminary evidence regarding the reliability of IJV ultrasound image acquisition by nurses without previous clinical ultrasound experience. Following preparatory training, the nurses were able to acquire IJV ultrasound images with good-to-excellent intra-operator and moderate-to-excellent inter-operator relative reliability. These findings support the technical feasibility of nurse-performed IJV ultrasound image acquisition under standardized conditions. Future studies involving more nurse operators and a larger, more diverse participant population, including patients with heart failure, should evaluate the reliability of nurse-performed IJV ultrasound image acquisition under real-world clinical conditions to further assess the reproducibility and generalizability of these findings and establish the feasibility and clinical validity of this approach.

## Figures and Tables

**Table 1:** Demographic characteristics of the participants (n = 17).

	Mean ± SD
Age (year)	19.9 ± 1.3
Body mass (kg)	51.1 ± 5.7
Height (cm)	160.0 ± 5.7
Body mass index (kg/m^2^)	20.0 ± 2.0

**Table 2:** Internal jugular vein diameter measurements derived from images acquired by the two operators.

Measurement Condition	Diameter	Operator A (mm)	Operator B (mm)
Min diameter	Major	13.4 ± 2.6	12.8 ± 2.7
Quiet breathing	Minor	7.5 ± 2.2	7.6 ± 2.4
Max diameter	Major	14.8 ± 2.7	14.1 ± 2.7
Quiet breathing	Minor	8.7 ± 2.0	8.9 ± 2.2
Max diameter	Major	17.8 ± 4.1	16.9 ± 3.9
Valsalva maneuver	Minor	11.1 ± 2.4	11.9 ± 2.3

Values are presented as mean ± SD.

**Table 3:** Intra-operator reliability of internal jugular vein ultrasound image acquisition based on diameter measurements.

Measurement Condition	Diameter	Operator A	Operator B
		ICC (1, 1)	ICC (1, 1)
Min diameter	Major	0.781	0.931
Quiet breathing	Minor	0.791	0.907
Max diameter	Major	0.834	0.935
Quiet breathing	Minor	0.765	0.911
Max diameter	Major	0.967	0.941
Valsalva maneuver	Minor	0.875	0.881

ICC (1, 1) represents intra-operator reliability based on a single-measurement, one-way random-effects model. ICC, intraclass correlation coefficient.

**Table 4:** Inter-operator reliability of internal jugular vein ultrasound image acquisition based on diameter measurements.

Measurement Condition	Diameter	1st	2nd	3rd
		ICC (2, 1)	ICC (2, 1)	ICC (2, 1)
Min diameter	Major	0.699	0.842	0.800
Quiet breathing	Minor	0.849	0.832	0.642
Max diameter	Major	0.799	0.887	0.916
Quiet breathing	Minor	0.878	0.855	0.683
Max diameter	Major	0.856	0.838	0.903
Valsalva maneuver	Minor	0.863	0.629	0.783

ICC (2, 1) represents inter-operator reliability based on a single measurement. ICC, intraclass correlation coefficient.

**Table 5:** Bland–Altman analysis of inter-operator agreement for internal jugular vein diameter measurements.

Measurement Condition	Diameter	Bias (mm)	95% LoA (mm)	95% CI of the Bias (mm)	*p* Value for Proportional Bias
Min diameter	Major	0.68	−1.78 to 3.13	0.03 to 1.37 *	0.78
Quiet breathing	Minor	−0.19	−2.63 to 2.25	−0.82 to 0.42	0.45
Max diameter	Major	0.72	−0.93 to 2.38	0.29 to 1.11 *	0.90
Quiet breathing	Minor	−0.21	−2.01 to 1.59	−0.66 to 0.26	0.44
Max diameter	Major	0.84	−2.55 to 4.23	−0.07 to 1.67	0.63
Valsalva maneuver	Minor	−0.77	−3.14 to 1.60	−1.42 to −0.18 *	0.86

Bias was calculated by subtracting the measurement derived from the image acquired by Operator A from that derived from the image acquired by Operator B. An asterisk (*) indicates fixed bias, defined as a 95% confidence interval of the bias that does not include zero. Proportional bias was assessed using linear regression of the differences between measurements derived from images acquired by the two operators against their corresponding means.

## Data Availability

The data supporting the findings of this study are available from the corresponding author upon reasonable request. The data are not publicly available due to participant privacy and ethical restrictions.

## Abbreviations

The following abbreviations are used in this manuscript:

CCA	Common carotid artery
ICC	Intraclass correlation coefficient
IJV	Internal jugular vein
IVC	Inferior vena cava
POCUS	Point-of-care ultrasound
SCM	Sternocleidomastoid muscle
SD	Standard deviation
